# Supplementary material for: Association of Sedentary Behavior with Brain Structure and Intelligence in Children with Overweight or Obesity: The ActiveBrains Project
Source: J Clin Med. 2020 Apr 12;9(4):1101. doi: 10.3390/jcm9041101 (PMC7230478; doi:10.3390/jcm9041101)
Supplement: Supplementary file 1 [file jcm-09-01101-s001.pdf]

Supplementary table 1. Brain regions showing negative associations between sedentary behaviors and gray matter volume in overweight/obese children after adjustment for total physical activity (n=99).

Brain Regions (mm³)	***x***	***y***	***z***	***t***	***Cluster size***
Watching TV					
Middle frontal gyrus	38	44	9	-5.37	201
Inferior frontal gyrus, pars triangularis	-39	20	27	-3.85	88
Inferior parietal gyrus	-53	-36	41	-3.66	61
Lingual gyrus	11	-87	-11	-4.45	354
Calcarine cortex	24	-59	12	-	-
Postcentral gyrus	-63	-12	11	-3.50	60
Playing video games					
Fusiform gyrus	33	-14	-33	-3.83	432
Inferior temporal gyrus	62	-24	-30	-3.74	270
Inferior temporal gyrus	-54	-30	-21	-4.05	300
Total sedentary time					
Cerebellum Crus I	20	-81	-27	-3.46	515
Cerebellum Crus II	-42	-60	-42	-3.93	2283

Analyses were adjusted for sex, peak height velocity offset (years), parent education university level (neither/one/both), body mass index (kg/m^2) and total physical activity assessed by YAP-S. All contrasts were thresholded using AlphaSim at $P < 0.001$with k=56 for watching TV, k=43 for playing video games, and k=56 for total sedentary time and surpassed Hayasaka correction. Anatomical coordinates (x, y, z) are given in Montreal Neurological Institute (MNI) Atlas space. No brain regions showed a statistically significant positive association between any sedentary behavior variable and gray matter volume.
